# A Clinical Evaluation of the Role of Autoimmunity in the Relation Between Erosions and Bone Mineral Density in Rheumatoid Arthritis

**DOI:** 10.3390/biomedicines12102376

**Published:** 2024-10-17

**Authors:** Margaux Moret, Caroline Morizot, Marcelo de Carvalho Bittencourt, Edem Allado, Isabelle Chary-Valckenaere, Damien Loeuille

**Affiliations:** 1 Department of Rheumatology, Nancy University Hospital, F-54000 Nancy, France; c.morizot@chru-nancy.fr (C.M.); i.valckenaere@chru-nancy.fr (I.C.-V.); d.loeuille@chru-nancy.fr (D.L.); 2 Department of Immunology, Nancy University Hospital, F-54000 Nancy, France; m.decarvalho@chru-nancy.fr; 3 ImoPA, UMR 7365 CNRS University of Lorraine, F-54000 Nancy, France; 4 University Center of Sports Medicine and Adapted Physical Activity, Nancy University Hospital, University of Lorraine, DevAH, F-54000 Nancy, France; e.allado@chru-nancy.fr

**Keywords:** rheumatoid arthritis, erosion, bone mineral density, autoimmunity, ACPA

## Abstract

**Highlights:**

What are the main findings?

- Anti-citrullinated peptide antibody (ACPA) titer is associated with a lower bone mineral density (BMD) in the hip, and an ACPA-positive status is associated with a higher erosion score
- Erosions are associated with a lower BMD and T-score in the hip and spine
- The relation between erosions and hip BMD is not found to be driven by autoimmunity

What are the implications of the main findings?

- The presence of ACPAs or erosions should lead to osteoporosis screening in rheumatoid arthritis patients

**Abstract:**

**Background/objectives:** Both erosions and osteoporosis in rheumatoid arthritis (RA) have common mechanisms. The aim of this study was to evaluate the relationship between erosion and bone mineral density (BMD) in RA and whether it can be driven by autoimmunity. **Methods:** Patients fulfilling the ACR 1987- or ACR/EULAR 2010-criteriae for RA. performed radiographs (erosions evaluated by the modified Sharp/van der Heidje erosion score) and biology for anti-citrullinated peptide antibodies (ACPAs), rheumatoid factors (RFs) and anti-nuclear antibodies (ANAs) at intervals of less than 2 years from dual-energy X-ray absorptiometry (DXA) for BMD assessment. **Results:** A total of 149 patients were included, (75.8% women, mean age of 62 y.o (SD 9.61) and a median disease duration of 132 months [60; 240]). A total of 61.1% patients were ACPA positive, 79.9% were erosive and 10.7% had a hip or spine T-score ≤ −2.5. A higher erosion score was associated with a lower BMD (value: −0.222; *p* = 0.009) and T-score (value −0.397; *p* < 0.0001) in the hip. ACPA status was associated with a higher erosion score (63.0 (53.2) vs. 45.5 (44.1) for ACPA- (*p* = 0.04)). ACPA titers were associated with a lower BMD in the hip (value −0.216; *p* = 0.01). In linear regression, erosion and BMD were still associated, but this association is not driven by ACPA status or titer. **Conclusions**: In RA patients, erosions and BMD are inversely associated but this relationship does not seem to be driven by autoimmunity only. However, the presence of ACPA or erosion should lead to osteoporosis screening.

## 1. Introduction

Rheumatoid arthritis (RA) is the most frequent chronic inflammatory rheumatism, with a prevalence of 1% worldwide [1,2]. It is characterized by the presence of local and general inflammation and peripheral articular destruction combining joint space narrowing and erosion. Erosions are secondary to osteoclastic activation, partly induced by proinflammatory cytokines (IL1beta, IL6, IL8, TNF-alpha) expressed in synovitis [3,4]. It is well known that erosions are correlated with the presence and titer of anti-citrullinated peptide antibodies (ACPAs) on radiographs but also on ultrasonography examinations (USs) [5,6,7,8]. The number and severity of erosions are greater in ACPA-positive RA than in ACPA-negative RA patients based on the modified Sharp/van der Heidje erosion score (SHSe) evaluation [5,7,8].

Rheumatoid arthritis is also known to be an independent factor of osteoporosis [9,10] with a twofold increased risk of vertebral and nonvertebral fracture compared to the general population [11,12,13]. Disease activity, disease duration, biological inflammation, smoking and corticosteroid intake are all risk factors contributing to bone loss and osteoporosis in RA patients [12]. Currently, the gold standard for the diagnosis and monitoring of osteoporosis is dual-energy X-ray absorptiometry (DXA) performed on the spine and hip. The prevalence of densitometric osteoporosis in RA varies from 10 to 50% according to the literature [14,15,16]. Additionally, it has already been demonstrated that ACPA titer or status are “significant predictors of bone mineral density (BMD) changes in the proximal femur” [17]. Moreover, an ACPA-positive status is associated with a lower BMD in the spine and hip even in early RA [18,19]. ACPAs are also associated with a higher likelihood of having a major fracture at 10 years or a hip fracture according to the FRAX^®^ [20].

The pathophysiology of erosion and bone loss is individually related to different pathways in ACPA-positive patients, such as direct activation of preosteoclast cells or immune cells (monocytes, macrophages, etc.) by ACPAs due to the presence of citrullinated peptides on their surface. As a consequence, TNF-alpha and receptor activator of nuclear factor kappa B ligand (RANK-L) synthesis are increased, leading to osteoclast differentiation and activation a second time [21,22,23]. At the local level, ACPAs increase RANK-L and proinflammatory cytokine expression in RA-fibroblast-like synoviocytes involved in synovitis [24]. Serum RANK-L is reported to be increased in ACPA-positive patients independent of acute-phase reactants and proinflammatory cytokines, thus affirming the role of autoimmunity passing through ACPAs themselves in local osteoclastogenesis [18]. Other mechanisms via OPG/Wnt/DKK1/sclerostin pathways have also been described [16].

Both erosion and osteoporosis in RA are related to osteoclast activation via RANK-L pathway stimulation. However, the role of autoimmunity, especially ACPA, in the association between erosion and bone mineral density has only sparsely been studied [25]. The primary objective of our study was to evaluate if there is an association between erosion and bone mineral density in RA. The secondary objectives were to evaluate whether autoimmunity parameters (status or titer), such as ACPAs, rheumatoid factors (RFs) and anti-nuclear antibodies (ANAs), might have a role in this association.

## 2. Materials and Methods

### 2.1. Study Population

Patients followed for rheumatoid arthritis (RA) in our department from January 2008 to May 2019 were selected for this monocentric cross-sectional study based on clinical, biological and imaging data performed in daily practice. The diagnosis of RA was defined by the 1987 ACR criteria or 2010 ACR-EULAR criteria for the most recent patients. To be included, patients had to undergo hand/foot radiographs and biology at intervals of less than 2 years from DXA. Then, only patients with continuous quantitative values of ACPA titers were included.

First, 1145 RA patients were selected. Among them, 867 patients did not undergo biology and X-rays at intervals of ≤2 years from DXA, and 129 patients did not have continuous quantitative values of ACPA titers. In total, we included 149 patients in the final analysis.

### 2.2. Clinical Data

Data were collected from the computerized medical records of our department: sex, age, BMI, tobacco use, disease duration, prior treatments (anti-osteoporotic, corticosteroids, DMARDs) and disease activity based on DAS28CRP.

### 2.3. Biological and Immunological Data

Levels of C-reactive protein (CRP) and erythrocyte sedimentation rate (ESR) were collected. The presence of inflammation was defined as a CRP value > 5 mg/L.

Immunological status and antibody titers (ACPA, RF, ANA) were extracted from the internal laboratory information system database of the Department of Immunology. From 2008 to June 2011, anti-CCP2 dosage kits (Werfen, 93310 Le Pre Saint Gervais, France) were used in our center with a positive threshold for ACPA titers ≥ 25 IU/L. ACPA titers are presented as continuous quantitative values. From July 2011, anti-CCP3 dosage kits (Werfen, France) were introduced. The threshold was also 25 IU/L, but continuous values were only available until 250 IU/L, and higher titers were reported as “>250 IU/L”. As ACPA titers are correlated with inflammation and erosions, we decided to exclude patients without a continuous titer of anti-CCP, which means all patients with a titer expressed as “>250 IU/L”. For RF dosage, only IgM serotypes were considered, and titers were given in quantitative values with a positivity threshold of 20 IU/L. The ANA titer threshold was set as ≥16 IU/L.

### 2.4. Dual-Energy X-ray Absorptiometry (DXA) Assessment

Dual-energy X-ray absorptiometry (DXA) data (Advance PA + 301010, ncore, version 14.10.022, Madison, WI, 53718, USA) were extracted from reports registered in our computerized database. The reproducibility of DXA measurements is known to be good [26,27]. We selected the T-score, Z-score and bone mineral density in g/cm^2^ in the lumbar spine (L1–L4) and proximal femur (i.e., hip).

The presence of osteoporosis on DXA was defined by a T-score ≤ −2.5, and the values were also expressed as continuous variables. We took the T-score (as a quantitative value) and BMD in the hip as the primary endpoints, as they are criteria for the FRAX^®^ score (Osteoporosis Research Ltd., London, UK).

### 2.5. Erosion Assessment

The assessment of erosions was performed by one reader (ICV) according to the modified Sharp/van der Heijde erosion score (SHSe) on radiographic posteroanterior views of hands and anteroposterior views of feet. Readings were performed blinded to the patient’s clinical and biological information. An erosion with a grade ≥2 was considered significant, and RA was considered erosive according to the EULAR 2013 definition [28], i.e., presence of erosion on at least three separate joints among the following sites: proximal interphalangeal joints (PIPs), metacarpophalangeal joints (MCPs), wrist (counted as one joint) and metatarsophalangeal joints (MTPs) on radiographs of both hands and feet [29]. The total SHSe score and their subscores for hand and feet were calculated. We considered the total SHSe score and erosive status to be primary endpoints.

### 2.6. Statistical Analysis

The distribution of the variables was assessed by the Shapiro–Wilk normality test. Among the variables, only age, DAS28CRP and T-score followed a normal distribution.

Qualitative variables are shown as number and percentage. Quantitative variables are shown as medians (first and third quartiles) in the case of an abnormal distribution and as the mean and standard deviation for normally distributed variables. In the univariate analysis, for normally distributed variables, we used a Student’s *t*-test for quantitative variables and the Mann–Whitney U test for qualitative variables. Among the other variables, we tested the association between two quantitative variables by using linear regression. For qualitative variables, the Fisher test was used. Only variables with a *p* value ≤ 0.1 in the univariate analysis were included in the multivariate analysis, and a multiple linear regression was performed. The results were assessed at the 95% confidence interval, and a *p* value < 0.05 was regarded as statistically significant. All statistical analyses were performed using XLSTAT version 2020.3.13^®^.

According to ethical considerations of our institution all patients were informed during their stay in our unit that data collected might be used for research purposes. No patient included in this study expressed any objection to the use of their personal data for research and publication so the data were extracted from regular medical records and anonymized for analysis. This study was recorded in the clinical research department according to the following number: 2020PI083.

## 3. Results

### 3.1. Patients Characteristics

**Figure 1:**
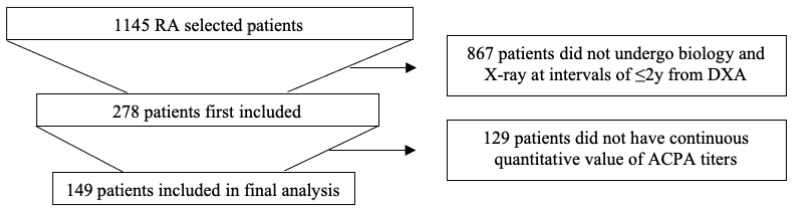
Flowchart of the study.

One hundred and forty-nine patients fulfilled the inclusion criteria (Figure 1). Patients characteristics are presented in Table 1. There was a majority of women (75.8%), and the patients’ mean age was 62 (SD 9.61) years old. Additionally, there was a relatively long duration of RA, with a median disease duration of 132 [60; 240] months. Most of them had moderate disease activity with a mean of 4.64 (SD 1.3) for DAS28CRP, and 79.8% had current or past corticosteroid therapy. Smoking was recorded in 20.1% of the patients. In total, at least 83.9% of the patients had at least one risk factor for osteoporosis added to RA.

The mean T-scores were −1.12 (SD 1.25) and −0.78 (SD 1.54) in the hip and spine, respectively. Sixteen patients (10.7%) had osteoporosis (i.e., T-score ≤ −2.5) in the hip, and sixteen patients (10.7%) had osteoporosis in the spine. Twenty-six (17.5%) patients were classified as osteoporotic at least at one site (Table 1). Past or current anti-osteoporotic treatment was administered in 31.5% of the patients. A large proportion of patients (79.9%) had an erosive disease (Table 1). The median SHSe total score was 40.0 [15; 81]. ACPAs and RFs were positive in 61.1% and 57% of the RA patients, respectively.

ACPA-positive RA patients had a significantly higher disease activity index than ACPA-negative RA patients (DAS28CRP of 4.8 (SD 1.3) vs. 4.3 (SD 1.2); *p* = 0.02). They were also more numerous, having RFs or ANAs with significantly higher titers than ACPA-negative patients. The total SHSe score was higher in ACPA-positive RA patients, with a median of 49.5 [20.7; 93.5] versus 29 [12; 65] (*p* = 0.04) in ACPA-negative patients. Details are presented in Table 1.

### 3.2. Erosion Assessment

In the univariate analysis, ACPA-positive patients had a significantly higher SHSe total score, with a mean score of 63.08 (SD 53.25) versus 45.55 (SD 44.06) in ACPA-negative RA (*p* = 0.04) (Table 2). No association was demonstrated between erosive status and ACPA status or titers (*p* value = 0.12). Significantly higher SHSe total scores were observed in RF-positive patients than in RF-negative patients: mean of 65.4 (SD 54.0) vs. 43.6 (SD 42.7), respectively (*p* = 0.016). Double positivity (ACPA positive and RF positive) was significantly associated with erosive RA, with a prevalence of 90.5% vs. 74.7% (*p* value = 0.044). Nevertheless, titers of autoantibodies had no significant relation with total SHSe.

SHSe total score and SHSe subscores were inversely associated with BMD and T-score in the hip, with the hip T-score determining approximately 16% of the SHSe total score (R^2^ = 0.158) (Table 2). Similar trends were observed for BMD and T-score in the spine (Table 2). The status of erosive disease was significantly associated with a lower hip BMD (0.831, SD 0.22 vs. 0.965, SD 0.16; *p* < 0.0001). The status of erosive disease was also significantly associated with a lower hip T score of −1.33 (SD 1.1) vs. −0.35 (SD 1.3) (*p* < 0.0001) for non-erosive RA. Similar trends were also observed in spine BMD and T-score.

Disease duration explained 31.5% of the SHSe total score variation (R^2^ = 0.315). Smokers had a significantly lower total SHSe than non-smokers: 38.4 (SD 47.7) vs. 56.8 (SD 49.2), respectively (*p* = 0.031). BMI was inversely associated with SHSe total score; for each increase of 1 unit of BMI, there was a decrease of −0.243 for the SHSe total score (value −0.243; *p* = 0.019).

**Figure 2:**
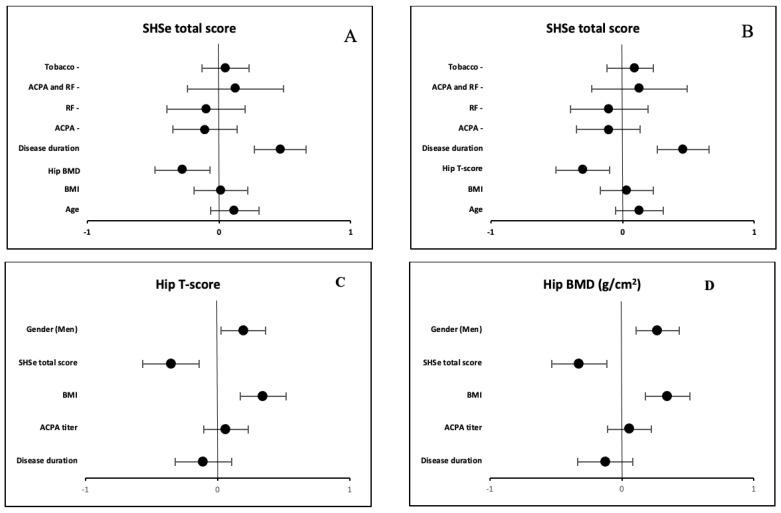
**Variables associated with SHSe total score, HipBMD and Hip T-score on multivariate analysis presented in forest plots.** (**A**) SHSe total score and associated variables with hip BMD included in the multivariate analysis. (**B**) SHSe total score and associated variables with hip T-score included in the multivariate analysis. (**C**) Hip T-score and associated variables. (**D**) Hip BMD and associated variables. SHSe: modified Sharp/van der Heijde erosion score, BMI: body mass index, BMD: bone mineral density, ACPA: anti-cyclic citrullinated peptide antibody, RF: rheumatoid factor. Linear regression was used. All variables included in the multivariate presented a *p*-value < 0.1 in the univariate analysis. The x-axis represents the estimated coefficient of regression.

In the multivariate analysis (Figure 2), disease duration and hip BMD were independent factors for erosion severity evaluated on the SHSe total score. When hip T-score was included in the linear regression model instead of hip BMD, the variables associated with the SHSe total score were disease duration and hip T-score (Figure 2). When the disease duration was excluded from the model, osteoporosis parameters (hip BMD and T-score) were still associated with the SHSe total score.

### 3.3. Bone Mineral Density and T-Score Assessment

The presence of ACPAs was not significantly associated with hip or spine BMD (Table 3 and Table 4, respectively). However, higher titers of ACPAs were associated with a lower BMD in the hip (value = −0.216; *p* = 0.01) but not in the spine. The presence of RFs or ANAs did not highlight any significant relation with either hip or lumbar spine BMD or T-score.

For the other demographic and clinical variables, women had a significantly lower BMD and T-score at the two sites and were significantly more often classified as osteoporotic in the hip (*p* value = 0.013) (Table 3). BMI was positively associated with DXA parameters at both sites, i.e., patients with a higher BMI had higher BMD or T-score. The intake or dose of corticosteroids were not associated with lower values of BMD and T-score in the hip and spine. Disease duration had a significant impact on BMD and T-score in the hip and explained 12.6% of the variation in the hip T-score (R^2^ = 0.126). These associations were also observed for spine BMD (Table 4). Osteoporosis in the hip was associated with higher disease activity scores, with a mean DAS28CRP of 5.5 (SD 1.6) versus 4.6 (SD 1.2) in patients without osteoporosis (*p* = 0.007) (Table 3). The same results were observed for spine osteoporosis (Table 4).

As previously shown, hip BMD and T-score were inversely associated with the SHSe total score. RA patients with an erosive disease also had lower hip BMD and hip T-scores (Table 2 and Table 3). Similar results were observed for the spine BMD and T-scores (Table 4).

In the linear regression model with hip BMD as the primary outcome, BMI, sex and SHSe total score were still independently associated with hip BMD. When erosive status was included in the model instead of SHSe total score, hip BMD was still independently associated with BMI, sex and erosive disease (Figure 2D). When hip T-score was the primary outcome of the linear regression model, BMI, gender and SHSe total score were independently associated with hip T-score. When erosive status was included instead of SHSe total score, hip T-score was still independently associated with BMI, sex and erosive disease (Figure 2C).

## 4. Discussion

Our aim was to determine for the first time whether bone fragility on the axial skeleton assessed by DXA (BMD and T-score in the hip) and erosion on peripheral joints evaluated by SHSe total score may be driven by ACPAs and/or other autoimmunity-related antibodies (RFs and ANAs). We found that erosions (based on SHSe score) were associated with a lower BMD and T-score in the hip but also the spine. This association was confirmed at the patient level when erosive status (at least three erosive joints) was evaluated. Nevertheless, our study failed to demonstrate a role of ACPAs (status or titers) in this association.

The major factors for erosion (SHSe total score) were disease duration, hip T-score and BMD. This last association was also described by Bruno et al. in a cohort of early-RA patients (n = 128) [15]. The exclusion of disease duration did not change the results, and hip T-score or BMD included separately were still significant independent factors for erosion severity. The univariate analysis showed that BMI and tobacco use were associated with lower erosion scores. Concerning BMI, Rydell et al. showed, in a population of 233 early-RA patients (<12 months of disease duration), that a high BMI might reduce the risk of severe joint damage assessed also by the modified Sharp–van der Heijde score [30]. In the Espoir cohort, Vesperini et al. showed that smokers had reduced radiographic progression at one year [31]. The variables associated with joint erosion on the modified Sharp–van der Heijde erosion score in the univariate analysis were the presence of ACPAs, RFs and double positivity. These relations are already known and previously reported in the literature [8].

In our study, 17.5% had osteoporosis in the hip and/or spine based on the DXA assessment. This result is consistent with the literature; indeed, the described prevalence of densitometric osteoporosis ranges from 10 to 50% [15,32]. A large proportion of the patients included in this study presented at least one osteoporotic risk factor (84%), and 31.5% were treated or had been treated with at least one anti-osteoporotic treatment. As previously reported, we showed that hip BMD or hip T-score were significantly associated with BMI and sex in the multivariate analysis. Women had lower BMD and T-scores in the hip, and BMI is known to be protective against bone loss [15].

We showed that ACPA titers and not status are associated with lower BMD but were not found to be independent factors in the multivariate analysis. This result is interesting as we know high titers of ACPAs are bad prognosis factors for RA [33]. In 578 early-RA patients, Llorente et al. found that ACPA-positive RA patients were significantly associated with a lower BMD in the hip and spine in both univariate and multivariate analyses [19]. Orsolini et al. evaluated only the Z score at both sites (spine and hip) in 127 RA patients. They failed to demonstrate any correlation between ACPA status and Z-score at the total femur, femoral neck or spine [34], but some associations were observed according to the threshold of positivity when the analysis was performed on quartiles. Interestingly, Orsolini et al. did not demonstrate any association between Z-score and RFs (status or titer). In a Dutch cohort (n = 408) of early-RA patients, Amkreutz et al. showed a lower BMD in the spine and hip of ACPA-positive patients than in ACPA-negative patients at the baseline, without the influence of ACPA levels and without significant changes over time. The difference in BMD did not reach the level of significance in the Swedish cohort (n = 198) at the baseline in ACPA patients (status or titers), and no change over time was noted between the two subsets. Concerning RFs, in the Dutch cohort, BMD was lower in the spine of RF-positive patients than RF-negative patients at the baseline in the univariate analysis. This was not confirmed in the multivariate analysis, and there was no significant variation over time [35]. Finally, Bruno et al. showed that osteopenia and osteoporotic status were associated with ACPA status in a population of early-RA patients (n = 71) only in the univariate analysis [15].

Autoimmunity seems to be less preponderant in long-duration RA. Indeed, in an established population of 149 RA patients—not early rheumatisms—we did not find any relation driven by ACPAs. In summary, the role of autoimmunity seems to be modest to explain the relation between erosions and systemic bone mineral density, and the role of ACPAs at disease onset should be clarified in further studies. These results may be related to the size of the sample and the proportion of patients with an ACPA-positive status.

The strength of our study is a satisfying proportion of ACPA-positive RA patients (61.8%), a large proportion of patients with erosive status (almost 80%) and a large range of SHSe total scores. Also, the assessment of erosive status besides SHSe score is particularly interesting for daily practice, as it is easy to determine if the patient has at least three eroded joints, whereas SHSe score is more time-consuming and suitable for clinical trials. Additionally, data for imaging (X-ray, DXA), biology (status and titers) and clinical variables were recorded within no more than 2 years.

The limitations of our study are the retrospective design with missing data and the exclusion of 129 patients without continuous titers of ACPAs, which reduced the sample size to 149 patients. This possibly led to an insufficient number of patients to show a relation with ACPA titers in the multivariate analysis. Also, we only studied autoimmunity based on the presence of autoantibodies and did not analyze immune cells such as the autoreactive B-cells providing those autoantibodies nor cytokine pathways (proinflammatory interleukins, TNF alpha, Janus kinase.). The involvement of these immune cells and cytokine pathways should be further investigated [36], not only in patients with established RA but also in pre-RA patients. Indeed, we know that autoantibodies such as ACPAs are present years before clinical onset [37]. These patients could then present bone changes before clinical onset, and these mechanisms could help to understand the role of autoimmunity in the relationship between local and systemic bone damage.

## 5. Conclusions

In summary, ACPA titer is associated with a lower BMD in the hip, and an ACPA-positive status is associated with a higher erosion score. Erosions and severity are associated with a lower BMD and T-score in the hip and spine. However, we have shown that the relationship between erosion and bone mineral density in RA does not seem to be driven by ACPAs or other autoimmunity-related antibodies but rather by disease duration. In clinical practice, erosive RA should lead to osteoporotic screening to initiate as soon as possible anti-osteoporotic treatment in RA patients with bone fragility risks. Further studies should also focus on analyzing immune cells or cytokine pathways.

## Figures and Tables

**Table 1:** Patient characteristics.

Variables	TOTAL (n = 149)	ACPA + (n = 91)	ACPA − (n = 58)	*p*-Value
Demographic and Clinical Characteristics				
Age (years)	62.1 (9.6)	61.5 (9.6)	63.1 (9.6)	NS
Women (%)	113 (75.8)	70 (76.9)	43 (74.1)	NS
Disease duration (months)	132 [60; 240]	156 [84; 240]	114 [36; 234]	NS
DAS 28CRP	4.6 (1.3)	4.8 (1.3)	4.3 (1.2)	0.020
BMI	26.1 [0; 31.6]	25.6 [22.1; 27.9]	26.5 [23.5; 33.3]	NS
Tobacco—currently (%)	30 (20.1)	16 (17.6)	14 (24.1)	NS
Treatments				
Corticosteroids: prior/current intake (%)	118 (79.2)	71 (78.0)	47 (81.0)	NS
Corticosteroid dose (mg/d)	7.5 [0; 10]	7.5 [0; 10]	5 [4.5; 10]	NS
DMARDs: prior/current intake (%)	91 (61.1)	57 (62.6)	32 (55.2)	NS
Anti-osteoporotic treatment: prior/current intake (%)	47 (31.5)	25 (27.5)	22 (37.9)	NS
Biology				
ACPA titers	149.0 [0; 527]	334 [191;1105]	0 [0;1]	-
RF positive (%)	85 (57.0)	67 (73.6)	18 (31.0)	<0.0001
RF titers (IU/mL)	32 [0; 130]	82 [17; 198]	0 [0; 25]	<0.0001
ANA positive (%)	122 (81.9)	82 (90.1)	40 (69.0)	<0.0001
ANA titers	256 [40; 512]	256 [128; 1024]	128 [0; 256]	0.002
ACPA and RF positive (%)	67 (45.0)	-	-	-
CRP (mg/L)	9.9 [3.4; 20.0]	10.3 [3.4; 22.1]	9.6 [4.3; 17.2]	NS
ESR (mm at 1st hour)	29 [17; 44]	31 [16; 49]	27 [20; 38]	NS
Imaging				
Radiography				
Erosive RA (%)	119 (79.9)	76 (83.5)	43 (74.1)	NS
Nb of erosions ≥ grade 2	14 [5; 27]	16 [7; 30]	12 [3; 23]	0.057
SHSe total score	40 [15; 81]	49 [2; 93]	29 [12; 65]	0.040
SHSe hand score	21 [5; 47]	27 [6; 55]	18 [3; 38]	NS
SHSe foot score	18 [4; 36]	20 [8; 40]	11.5 [2; 28]	0.010
DEXA				
T-score in hip	−1.1 (1,3)	−1.2 (1.2)	−1.0 (1.3)	NS
T-score < −2.5 in hip (%)	16 (10.7)	9 (9.9)	7 (12.1)	NS
Z-score in hip	−0.1 (1.1)	−0.2 (1.0)	0.1 (1.2)	NS
BMD (g/cm^2^) in hip	0.9 [0.8; 1]	0.8 [0.8; 0.9]	0.9 [0.8; 1]	NS
T-score in lumbar spine	−0.8 (1.5)	−0.8 (1.5)	−0.8 (1.6)	NS
T-score < −2.5 in lumbar spine (%)	16 (10.7)	8 (8.8)	8.0 (13.8)	NS
Z-score in lumbar spine	0.3 (1.6)	0.3 (1.6)	0.4 (1.4)	NS
BMD (g/cm^2^) in lumbar spine	1.1 [0.9; 1.2]	1.1 [0.9; 1.2]	1.1 [0.9; 1.2]	NS
T-score < −2.5 in lumbar spine and/or hip (%)	26 (17.5)	8 (8.8)	9 (15.5)	NS

SD: standard deviation, Q1: first quartile, Q3: third quartile, csDMARD: conventional synthetic disease-modifying antirheumatic drug, ACPA: anticyclic citrullinated peptide antibody, RF: rheumatoid factor, ANA: anti-nuclear antibody, CRP: C-reactive protein, SHSe: modified Sharp/van der Heijde erosion score, Nb: number, NS: not significant, BMD: bone mineral density, d: day. A Student’s *t*-test, Mann–Whitney U-test, Khi2 or Fisher’s test were used to compare ACPA + and ACPA – patients data.

**Table 2:** Variables associated with erosions in the univariate analysis.

		SHSe Total Score			Erosive Status		
Qualitative Variables (n = 149)		Mean (SD)		*p* Value	Nb of Erosive RA (%)		*p* Value
Women	Yes	56.70 (51.55)		NS	90 (81.8)		NS
	No	52.82 (48.25)			29 (82.9)		
Tobacco use	Yes	38.44 (47.69)		0.031	17 (60.7)		0.005
	No	56.76 (49.20)			95 (86.4)		
Corticosteroid intake	Yes	54.32 (50.56)		NS	94 (82.5)		NS
	No	61.83 (50.84)			25 (80.6)		
ACPA	Yes	63.08 (53.25)		0.04	76 (86.4)		NS
	No	45.55 (44.06)			43 (75.4)		
RF	Yes	65.38 (54.29)		0.016	72 (87.8)		0.05
	No	43.63 (42.75)			47 (74.6)		
ANA	Yes	57.86 (52.76)		NS	96 (81.4)		NS
	No	50.17 (39.63)			22 (88.0)		
ACPA and RF	Yes	65.51 (54.55)		0.055	57 (90.5)		0.044
	No	48.90 (45.96)			62 (74.7)		
**Quantitative Variables** **(n = 149)**		**R^2^**	**Value**	**Pr > |t|**	**Mean (SD)**		***p* Value**
					**Erosive**	**Non-Erosive**	
Age		0.036	0.190	0.022	63.7 (9.2)	57.0 (9.3)	0.001
Disease duration		0.315	0.561	<0.0001	180.6 (126.1)	82.8 (64.9)	<0.0001
DAS 28CRP		0.003	0.056	NS	4.6 (1.4)	4.6 (0.8)	NS
BMI		0.059	−0.243	0.019	26.7 (5.5)	29.5 (8.3)	NS
Corticosteroid dose (mg/d)		0.002	0.045	NS	8.6 (9.3)	8.9 (11.3)	NS
CRP		0.001	0.024	NS	19.5 (26.2)	13.8 (18.6)	NS
ACPA titer		0.001	0.032	NS	480.8 (760.4)	431.2 (875)	NS
RF titer		0.000	0.011	NS	120.6 (179)	108.8 (278.5)	0.09
ANA titer		0.007	0.086	NS	929.3 (3175)	1960.7 (6601.6)	NS
Hip BMD		0.049	−0.222	0.019	0.8 (0.2)	1.0 (0.2)	<0.0001
Hip T-score		0.158	−0.397	<0.001	−1.3 (1.1)	−0.3(1.3)	0.001
Spine BMD		0.032	−0.178	0.033	1.2 (0.2)	1.0 (0.2)	0.038
Spine T-score		0.032	−0.178	0.033	−0.9 (1.4)	−0.1 (1.4)	0.010

SD: standard deviation, ACPA: anti-cyclic citrullinated peptide antibody, RF: rheumatoid factor, ANA: anti-nuclear antibody, CRP: C-reactive protein, BMI: body mass index, SHSe: modified Sharp/van der Heijde score, BMD: bone mineral density, Nb: number, NS: non-significant and *p* > 0.1, d: day. For quantitative variables, a Student’s *t*-test was used or linear regression to test the association between two quantitative variables (e.g., with SHSe total score). For qualitative variables, a Mann-Whitney U test or Fisher test were used depending on a normal distribution.

**Table 3:** Variables associated with bone mineral density and osteoporosis (T-score ≤ −2.5) at hip in the univariate analysis.

		HIP								
Qualitative Variables (n = 149)		BMD (g/cm^2^)			T-Score			T-Score (Binary)		
		Mean (SD)		*p* Value	Mean (SD)		*p* Value	OR [CI 95%]		
Women	Yes	0.849 (0.16)		0.003	−1.27 (1.27)		0.018	0.185 [0.033–1.031]		
	No	0.890 (0.36)			−0.69 (1.06)					
Tobacco use	Yes	0.877 (0.17)		NS	−1.12 (1.30)		NS	1.091 [0.305–3.896]		
	No	0.855 (0.24)			−1.12 (1.25)					
Corticosteroid intake	Yes	0.852 (0.23)		NS	−1.17 (1.21)		NS	0.794 [0.250–2.525]		
	No	0.894 (0.17)			−0.92 (1.41)					
Erosive status	Yes	0.835 (0.23)		0.001	−1.31 (1.18)		0.001	3.75 [0.664–21.182]		
	No	0.971 (0.16)			−0.40 (1.22)					
ACPA	Yes	0.842 (0.25)		NS	−1.20 (1.20)		NS	0.814 [0.293–2.257]		
	No	0.883 (0.16)			−1.05 (1.29)					
RF	Yes	0.831 (0.25)		NS	−1.28 (1.12)		NS	0.986 [0.356–2.730]		
	No	0.893 (0.17)			−0.97(1.35)					
ANA	Yes	0.843 (0.23)		NS	−1.23 (1.24)		NS	3.22 [0.569–18.267]		
	No	0.916 (0.14)			−0.73 (1.18)					
ACPA and RF	Yes	0.823 (0.27)		NS	−1.28 (1.09)		NS	0.734 [0.260–2.073]		
	No	0.888 (0.17)			−1.02 (1.33)					
**Quantitative Variables** **(n = 149)**		**BMD (g/cm^2^)**			**T-Score**			**T-Score (Binary)**		
		**R^2^**	**Value**	**Pr > |t|**	**R^2^**	**Value**	**Pr > |t|**	**Mean (SD)**		***p* Value**
								**T-sc ≤ −2.5**	**T-sc > −2.5**	
Age		0.002	−0.041	NS	0.039	−0.197	0.02	61.5 (9.8)	62.2 (9.6)	NS
Disease duration		0.052	−0.228	0.007	0.126	−0.355	<0.0001	201.2 (127.9)	151.3 (120.2)	NS
DAS 28CRP		0.013	−0.114	NS	0.005	−0.072	NS	5.5 (1.5)	4.6 (1.2)	0.007
BMI		0.218	0.467	<0.0001	0.220	0.469	<0.0001	73.3 (46.8)	51.1 (47.8)	NS
Corticosteroid dose (mg/d)		0.000	0.039	NS	0.000	0.012	NS	8.1 (11.0)	8.3 (8.4)	NS
CRP		0.002	−0.047	NS	0.017	−0.131	NS	32.6 (42.6)	16.1 (21.0)	NS
ACPA titer		0.047	−0.216	0.01	0.000	−0.009	NS	272.3 (452.2)	489.6 (787.8)	NS
RF titer		0.021	−0.146	0.084	0.017	−0.132	NS	143.3 (309.6)	117.0 (189.4)	NS
ANA titer		0.000	−0.015	NS	0.002	−0.050	NS	3303.2 (8227.8)	822.9 (3051.2)	NS
SHSe total score		0.049	−0.222	0.009	0.158	−0.397	<0.0001	79.4 (51.5)	50.6 (47.9)	NS

SD: standard deviation, T-sc: T-score, ACPA: anti-cyclic citrullinated peptide antibody, RF: rheumatoid factor, ANA: anti-nuclear antibody, BMD: bone mineral density, BMI: body mass index, SHSe: modified Sharp/van der Heijde erosion score, Nb: number, NS: non-significant (*p* > 0.05), d: day. For quantitative variables, a Student’s *t*-test was used or linear regression to test the association between two quantitative variables (e.g., with BMD (g/cm^2^) and T-score). For qualitative variables, a Mann-Whitney U test or Fisher test were used depending on a normal distribution.

**Table 4:** Variables associated with bone mineral density and osteoporosis (T-score ≤ −2.5) at lumbar spine in the univariate analysis.

LUMBAR SPINE										
Qualitative Variables (n = 149)		BMD (g/cm^2^)			T-Score			T-Score (Binary)		
		Mean (SD)		*p* Value	Mean (SD)		*p* Value	OR [CI 95%]		
Women	Yes	1.050 (0.19)		0.0007	−0.941 (1.62)		0.003	0.185 [0.033–1.031]		
	No	1.153 (0.14)			−0.291 (1.20)					
Tobacco use	Yes	1.100 (0.19)		NS	−0.778 (1.50)		NS	0.495 [0.121–2.018]		
	No	1.068 (0.19)			−0.843 (1.56)					
Corticosteroid intake	Yes	1.073 (0.18)		NS	−0.816 (1.46)		NS	0.540 [0.179–1.624]		
	No	1.080 (0.22)			−0.750 (1.86)					
Erosive status	Yes	1.055 (0.17)		0.033	−0.936 (1.43)		0.007	3.641 [0.645–20.558]		
	No	1.168 (0.23)			−0.004 (1.91)					
ACPA	Yes	1.076 (0.18)		NS	−0.849 (1.63)		NS	0.590 [0.214–1.627]		
	No	1.072 (0.20)			−0.760 (1.48)					
RF	Yes	1.062 (0.17)		NS	−0.965 (1.26)		NS	0.714 [0.260–1.964]		
	No	1.096 (0.20)			−0.667 (1.68)					
ANA	Yes	1.069 (0.19)		NS	−0.835 (1.59)		NS	3.224 [0.569–18.267]		
	No	1.100 (0.15)			−0.687 (1.21)					
ACPA and RF	Yes	1.064 (0.18)		NS	−0.959 (1.24)		NS	0.698 [0.248–1.968]		
	No	1.082 (0.19)			−0.741 (1.61)					
**Quantitative Variables** **(n = 149)**		**BMD (g/cm^2^)**			**T-Score**			**T-Score (Binary)**		
		**R^2^**	**Value**	**Pr > |t|**	**R^2^**	**Value**	**Pr > |t|**	**Mean (SD)**		***p* Value**
								**T-sc ≤ −2.5**	**T-sc > −2.5**	
Age		0.001	−0.028	NS	0.001	−0.029	NS	61.7 (9.8)	62.1 (9.5)	NS
Disease duration		0.031	−0.177	0.032	0.002	0.043	NS	73.3 (46.8)	160.9 (127.9)	0.009
DAS 28CRP		0.002	−0.046	NS	0.007	0.085	NS	5.3 (1.6)	4.6 (1.2)	0.032
BMI		0.061	0.247	0.018	0.058	0.242	0.021	26.0 (4.2)	27.7 (6.9)	NS
Corticosteroid dose (mg/d)		0.000	0.011	NS	0.000	0.000	NS	6.7 (10.3)	9.1 (9.6)	NS
CRP		0.003	−0.053	NS	0.003	−0.059	NS	23.1 (32.9)	17.7 (23.8)	NS
ACPA titer		0.001	0.028	NS	0.033	−0.182	0.026	308.0 (409.1)	502.1 (816.8)	NS
RF titer		0.012	−0.110	NS	0.018	−0.135	NS	128.1 (177.9)	116.5 (206.0)	NS
ANA titer		0.014	−0.118	NS	0.001	−0.024	NS	2888.3 (8170.8)	878.8 (3069.5)	NS
SHSe total score		0.032	−0.178	0.033	0.032	−0.178	0.033	52.5 (43.6)	56.6 (51.5)	NS

SD: standard deviation, T-sc: T-score, ACPA: anti-cyclic citrullinated peptide antibody, RF: rheumatoid factor, ANA: anti-nuclear antibody, BMD: bone mineral density, BMI: body mass index, SHSe: modified Sharp/van der Heijde erosion score, Nb: number, NS: non-significant (*p* > 0.05), d: day. For quantitative variables, a Student’s *t*-test was used or linear regression to test the association between two quantitative variables (e.g., with BMD (g/cm^2^) and T-score). For qualitative variables, a Mann-Whitney U test or Fisher test were used depending on a normal distribution.

## Data Availability

The datasets used and/or analyzed during the current study are available from the corresponding author on reasonable request.

## Abbreviations

SD: standard deviation, T-sc: T-score, ACPA: anti-cyclic citrullinated peptide antibody, RF: rheumatoid factor, ANA: anti-nuclear antibody, BMD: bone mineral density, BMI: body mass index, SHSe: modified Sharp/van der Heijde erosion score, Nb: number, d: day.
